# The role of transforming growth factor-*β* (TGF-*β*) in the formation of exhausted CD8 + T cells

**DOI:** 10.1007/s10238-024-01394-0

**Published:** 2024-06-17

**Authors:** Rong Ma, Jin-Han Sun, Yan-Yang Wang

**Affiliations:** 1 Department of Radiation Oncology, General Hospital of Ningxia Medical University, Yinchuan, 750004 Ningxia China; 2 Institute of Medical Sciences, General Hospital of Ningxia Medical University, Yinchuan, 750004 Ningxia China; 3 Cancer Institute, General Hospital of Ningxia Medical University, Yinchuan, 750004 Ningxia China; 4 Graduate School, Ningxia Medical University, Yinchuan, 750004 Ningxia China

**Keywords:** CD8 + T cells, Exhaustion, TGF-*β*, Immunotherapy

## Abstract

CD8 + T cells exert a critical role in eliminating cancers and chronic infections, and can provide long-term protective immunity. However, under the exposure of persistent antigen, CD8 + T cells can differentiate into terminally exhausted CD8 + T cells and lose the ability of immune surveillance and disease clearance. New insights into the molecular mechanisms of T-cell exhaustion suggest that it is a potential way to improve the efficacy of immunotherapy by restoring the function of exhausted CD8 + T cells. Transforming growth factor-*β* (TGF-*β*) is an important executor of immune homeostasis and tolerance, inhibiting the expansion and function of many components of the immune system. Recent studies have shown that TGF-*β* is one of the drivers for the development of exhausted CD8 + T cells. In this review, we summarized the role and mechanisms of TGF-*β* in the formation of exhausted CD8 + T cells and discussed ways to target those to ultimately enhance the efficacy of immunotherapy.

## Introduction

Immunotherapy is emerging as a critical way to control disease [1, 2]. CD8 + T cells are the most important effector cells in the process of immunotherapy, and their numbers and functional status largely determine the success or failure of immunotherapy [3, 4]. However, under persistent antigen stimulation or inflammatory factors exposure, the function of CD8 + T cells gradually deteriorated and was in a state known as “exhaustion.” CD8 + T-cell exhaustion refers to a state of functional impairment or dysfunction observed in cytotoxic CD8 + T cells, which are crucial components of the adaptive immune response. These cells, also known as cytotoxic T lymphocytes (CTLs), play a pivotal role in recognizing and eliminating infected or malignant cells. The function of CD8 + T-cell exhaustion is complex and context-dependent. On the one hand, CD8 + T-cell exhaustion represents a host immune response mechanism to limit tissue damage caused by excessive inflammation and immune-mediated pathology. By dampening T-cell responses, exhaustion may prevent immunopathology in chronic infections or tumors. On the other hand, CD8 + T-cell exhaustion can contribute to immune evasion by pathogens or tumors, allowing them to persist and evade immune surveillance, which usually leads to out of control and progression of the disease [5–8]. However, reinvigoration of exhausted T cells by blocking inhibitory receptors can facilitate the improvement of immune function and the treatment outcome of disease [9]. Recent studies have demonstrated a significant correlation between exhausted T cells and patients’ response to immunotherapy. These exhausted T cells, especially the progenitor exhausted T cells, have become the key target for immunotherapy [10]. In-depth understanding of the biological characteristics of T cells exhaustion and molecular mechanisms of its formation will help to establish rational immunotherapeutic interventions.

Transforming growth factor-beta (TGF-*β*) is a multifunctional cytokine involved in various physiological processes, including cell growth, differentiation, apoptosis, and immune regulation. It is produced by a wide range of cell types, including immune cells (such as regulatory T cells, macrophages, and dendritic cells), stromal cells, and epithelial cells. TGF-*β* plays a pivotal role in maintaining immune homeostasis by orchestrating various processes such as the differentiation of T cells, dampening T-cell activation, promoting tolerance in antigen-presenting cells (APCs), and fine-tuning B-cell activity. Dysregulated production or signaling of TGF-*β* can contribute to the pathogenesis of various diseases, including cancer, fibrosis, autoimmune diseases, and inflammatory disorders [11–13]. A large number of studies have studied how TGF-*β* regulated the proliferation, differentiation, and localization of T cells [14]. At the same time, studies have also shown that TGF-*β* signal was a key approach to generate the exhausted CD8 + T cells [15–17]. Therefore, TGF-*β* signal is a window to explore the mechanisms of CD8 + T cells exhaustion.

Here, we reviewed the latest evidences of TGF-*β* in the formation of exhausted CD8 + T cells, with an emphasis on the underlying mechanisms of TGF-*β*-mediated T cells exhaustion. Their translational significances for the enhancement of immunotherapy efficacy and biomarkers for patient selection were also discussed. This review can deepen our understanding of the formation of exhausted CD8 + T cells, help to clarify the key role of exhausted CD8 + T cells in immunotherapy, and guide the development of novel immunotherapy for disease control.

## Exhausted CD8 + T cells

After exposure to persistent antigens and activated signals, such as during cancer development or chronic viral infection, CD8 + T cells developed into a state of terminal differentiation called T cells exhaustion [18]. The exhausted T cells are characterized by gradual loss of effector function, decreased ability of proliferation and differentiation, impaired memory and self-renewal, and changes in transcriptional and epigenetic processes with the upregulation of multiple inhibitory receptors. This means that after the differentiation process of exhaustion, T cells were locked in a state of permanent dysfunction, which hindered the immune response of virus-specific and tumor-specific T cells [19]. However, under certain conditions, the exhaustion status could be reversed. Reversing exhausted CD8 + T cells is one of the effective ways to reactivate immune function. Immune checkpoint inhibitors (ICIs) targeting programmed death-1 (PD-1) and its ligand PD-L1 were developed based on this property of exhausted CD8 + T cells. Antibody, which blocked the interaction of PD-1 and PD-L1, reversed the exhaustion phenotypes and restored T cells proliferative in chronic infection and cancer, thus improving the control of these diseases [10, 20]. Recent evidence showed that exhausted T cells were heterogeneous, including progenitor exhausted CD8 + T cells and terminally exhausted CD8 + T cells. Progenitor exhausted CD8 + T cells can generate a variety of cytokines and proliferate in vivo, and respond to PD-1 blockade through the expression of transcription factor T cytokine 1 (TCF1). However, terminally exhausted CD8 + T cell only produces single cytokine and upregulates granzyme B, and does not respond to PD-1 blockade [21–23].

The development of exhausted T cells was often considered a continuous differentiation process characterized by unique transcriptional mechanisms, gene expression profiles, metabolic changes, and epigenetic patterns [24, 25]. During the development of T cells exhaustion, sustained stimulation of T-cell receptors (TCR) activated transcription factor nuclear factor of activated T cells (NFAT) [26]. Subsequently, NFAT suppressed TCF1, promoted the expression of thymocyte selection-associated high mobility group box factor (TOX) [27, 28] and nuclear receptor 4A (NR4A) [29], and led to upregulation of multiple inhibitory immune checkpoint proteins, including PD-1, T-cell immunoglobulin and mucin domain-containing protein 3 (TIM-3), lymphocyte activation gene-3 (LAG-3), T-cell immunoglobulin and ITIM domain (TIGIT), and cytotoxic T lymphocyte antigen 4 (CTLA-4) [30–33]. However, the underlying mechanisms and activation processes that control the entry of T cells into an exhausted state are still largely unclear.

## TGF-*β* signaling pathway

TGF-*β* is a cytokine widely involved in cell growth, differentiation, homeostasis, angiogenesis, and immune response regulation [34–36]. In mammals, there are three subtypes of TGF-*β*: TGF-*β*1, TGF-*β*2, and TGF-*β*3. TGF-*β*1 is considered to play an important role in immune response regulation [37, 38].

The activation of TGF-*β* signaling pathway begins with the binding of TGF-*β* and TGF-*β* receptor II (TGF-*β*RII) chain, which can promote the recruitment and phosphorylation of the intracellular domain of TGF-*β*RII chain, and then initiates the downstream signaling pathway [39]. Smad2 and Smad3 are the main targets of TGF-*β* receptor kinase activity. Phosphorylation of Smad2 and Smad3 serine residues produces acidic tails required for oligomerization between their mad homology 2 (MH2) domains and Smad4. The Smad dimer or trimer is then transferred to the nucleus to regulate the expression of genes carrying the Smad response regulatory region. In addition to Smad4 pathway, R-Smads can also bind to transcriptional intermediary factor 1 *γ* (TIF1*γ*) or IκB kinase *α* (IKK*α*) protein directly and participate in TGF-*β* signaling transduction [40]. Furthermore, TGF-*β* also induces Smad-independent pathway through mitogen-activated protein (MAP) kinase, which transmits signaling via extracellular signal-regulated kinase 1/2 (ERK1/2) or TGF-*β*-activated kinase 1 (TAK1) [41].

TGF-*β* is demonstrated participated in the regulation of innate immune response and adaptive immune response widely. As far as T cells are concerned, TGF-*β* ensures the maintenance of T cells diversity and self-tolerance and initiates adaptive T cells immune response by regulating the development of thymic T cells and the survival, proliferation, and differentiation of peripheral T cells [36, 42]. Recent evidence shows that TGF-*β* is also a great contributor in promoting the formation of exhausted T cells [43].

## TGF-*β* is involved in the formation of CD8 + T-cell exhaustion

Recent studies have confirmed that there was a correlation between TGF-*β* and CD8 + T cells exhaustion, suggesting that TGF-*β* may be involved in the formation of CD8 + T cells exhaustion.

In cancer research, chronic exposure to TGF-*β*1, combined with persistent stimulation of TCR, was an effective way to establish exhausted CD8 + T cells in vitro [15]. The potential role of TGF-*β* in the development of exhausted CD8 + T cells was also demonstrated in in vivo studies. In cervical cancer, exhausted immune profile was identified by non-negative matrix factorization method in patient specimens. The exhausted subtype of cervical cancer was found expressed TGF-*β* response signature [44]. In urinary bladder cancer, higher frequency of exhausted CD8 + T cells was found in sentinel nodes. Exhausted T cells of urinary bladder cancer highly expressed PD-1 and GATA-binding protein 3 (GATA3). Proteomic analysis of the cell culture supernatant showed that TGF-*β*2 secreted by muscle invasive bladder cancer cells contributed to the formation of exhausted CD8 + T cells [45]. The role of miRNAs in the state of T cells exhaustion in acute lymphoblastic leukemia was explored. Of the total miRNAs analyzed, 10 were found to be associated with T cells exhaustion. Further analysis showed that these identified miRNAs were associated with genes related to TGF-*β*, the forkhead box O (FOXO), and MAPK signaling pathways [46]. Activin A is a member of the TGF-*β* superfamily [47]. Wiley et al. [48] found that activin co-localization promoted T cells exhaustion in the tumor microenvironment of colorectal cancer patients.

Furthermore, other studies have shown that blocking TGF-*β* can reverse the exhausted status of CD8 + T cells. Bone marrow (BM) CD8 + T cells of patients with multiple myeloma expressed high level of PD-1 and had the exhausted phenotype, and their proliferation and effector function were impaired. However, blocking PD-1 alone was not enough to reinvigorate the BM exhausted CD8 + T cells. Combined blocking of PD-1 and TGF-*β* could significantly promote the proliferation of BM exhausted CD8 + T cells from multiple myeloma patients [49]. Moreover, it has been found that exhausted CD8 + T cells could be reversed by T-cell-derived nanovesicles (TCNVs), which was closely related to the TGF-*β* receptor on the surface of TCNVs [50]. Bone morphogenetic protein (BMP) is a member of TGF-*β* family. Boosting BMP signaling while blocking TGF-*β*1 substantially increased polyfunctionality, while suppressing expression of exhaustion-related molecules and maintained the tumor killing ability of CD8 + T cells, enhanced tumor control in mice, and boosted exhausted T-cell responses to ICI therapy [15]. Glycoprotein-A repetitions predominant (GARP) is a cell surface docking receptor that activates latent TGF-*β*. PIIO-1 is a humanized anti-GARP monoclonal antibody that specifically binds to ligand-free GARP in tumor-infiltrating immune cells and blocks the formation of GARP-LTGF*β*1 complex. PIIO-1 reversed the exhaustion of T cells and overcame primary resistance to anti-PD-1 ICIs was demonstrated in recent study [51]. In aggressive B-cell non-Hodgkin lymphoma, galunisertib (TGF-*β* RI inhibitor) in combination with the chemotherapeutic agent doxorubicin exerted synergistic anticancer effects through preventing CD8 + T cells exhaustion [52]. In head and neck cancer, reversing exhausted CD8 + T cells via neutralizing TGF-*β* was one of the ways to improve the efficacy of neoadjuvant immunotherapy [53].

In the model of chronic viral infection, Hu et al. [16] revealed that TGF-*β* inhibited the differentiation of PD-1 + T-cell factor 1 (TCF-1) + stem cells into effector-like transitory subpopulation by maintaining the unique transcriptional process of CD8 + T cells, thus promoting the formation of exhausted CD8 + T cells. However, Tinoco et al. [54] found that TGF-*β* signal was not required for exhausted function in infection disease model. The dysfunction of CD8 + T cells induced by TGF-*β* was achieved by activating apoptosis.

## Molecular mechanisms of TGF-*β* promoted CD8 + T cells exhaustion

**Fig. 1:**
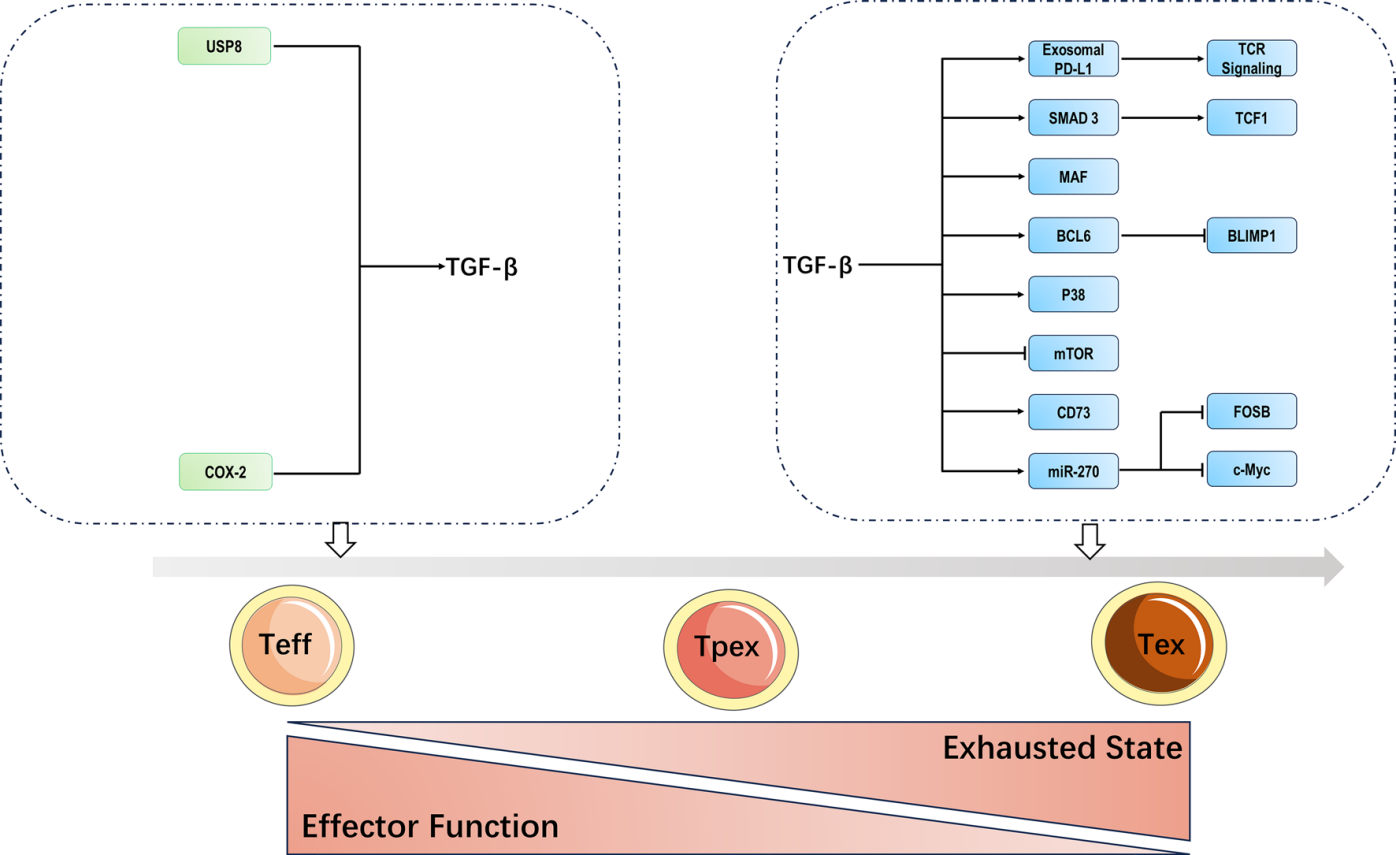
Molecular mechanisms of TGF-*β* promoted CD8 + T cells exhaustion

TGF-*β* can promote CD8 + T-cell exhaustion in both the tumor microenvironment and sentinel nodes/draining lymph nodes through various mechanisms, including local immunosuppression, induction of inhibitory receptors, promotion of regulatory T cells, and modulation of transcriptional programs (Fig. 1). PD-1/PD-L1 pathway is one of the ways to mediate immunosuppression [55]. TGF-*β* was shown to be the promoter of exosomal PD-L1. TGF-*β* increased the secretion of extracellular PD-L1 from breast cancer cells, which, in turn, mediated the exhaustion of CD8 + T cells by regulating the early phosphorylation of TCR signaling. This result was confirmed in in vivo studies. Inhibition of exosome release and TGF-*β* in vivo synergistically reduced tumor burden through reversing exhausted T cells and promoting the release of granzyme and interferon-gamma (IFN-*γ*) [56]. Also in breast cancer, the formation of exhausted T cells was thought to be associated with tumor-derived extracellular vesicles (TEV). TEV, which contained the central component of TGF-*β*, affected the exhaustion of T cells in tumor environment. TGF-*β* significantly upregulated the level of TβRII in TEV. The results of in vivo and in vitro studies showed that TEV with TβRII as cargo delivered to CD8 + T cells induced the activation of Smad3, which synergistically bound to T cells exhaustion associated genes with TCF1, resulting in CD8 + T cells exhaustion and consequently suppressing anticancer immunity [17]. In the meanwhile, studies from the same group have also shown that TGF-*β*, which promoted the exhaustion of CD8 + T cells in breast cancer, was regulated by ubiquitin-specific protease 8 (USP8). USP8 directly deubiquitinated and stabilized type II TGF-*β* receptor TβRII, resulting in increased expression of TGF-*β* receptor in plasma membrane and TEV. The increased secretion of TβRII + circulating extracellular vesicles (crEVs) enhanced the exhaustion of cytotoxic T cells. USP8 inhibitors could effectively antagonize the TGF-*β*/Smad signaling pathway in cancer cells, decrease the stability of TβRII and the number of TbRII + crEvs, thereby preventing tumor-induced exhaustion of CD8 + T cells and activating anticancer immunity, thus reducing cancer size and metastasis [57].

Maf is the basic leucine zipper transcription factor and belongs to the activator protein-1 (AP-1) superfamily [58]. Maf contributed to the formation of exhausted T cells was found in melanoma [59]. The overexpression of Maf made CD8 + T cells differentiated to exhausted phenotype. Maf-transduced CD8 + T cells expressed higher levels of PD-1 and lower levels of granzyme than mock-transduced T cells. Both IFN-*γ* and interleukin-2 (IL-2) produced by CD8 + T cells transduced with Maf were lower than those of un-transduced T cells. In contrast, Maf-deficient cancer-specific CD8 + T cells were much more capable of inhibiting cancer growth in vivo. Further study revealed that TGF-*β* increased the expression of Maf, IL-10, and B-cell lymphoma 6 (BCL6) in CD8 + T cells, and inhibited their accumulation in cancer and anticancer activity. The overexpression of BCL6 in CD8 + T cells induced by TGF-*β* was also found in head and neck cancers. BCL6 greatly improved the efficacy of anti-PD-1 therapy by antagonizing B-lymphocyte-induced maturation protein 1 (BLIMP1), inhibiting the expression of related genes in terminally differentiated CD8 + T (*T*_erm_) cells and inducing the expression of related genes in progenitor-like CD8 + T (*T*_prog_) cells [60].

Cyclooxygenase (COX)-2 had been found to be involved in the formation of T cells exhaustion in hepatocellular carcinoma (HCC). COX-2 promoted the exhaustion of CD8 + T cells through activating the TGF-*β* pathway. Elevated levels of phosphorylated Smad3, phosphorylated Samd2, and forkhead box protein 1 (FOXP1) were detected in exhausted T cells in HCC [61]. Furthermore, phosphorylated p38 was also identified as a downstream molecule of TGF-*β* and contributed to the development of T cells exhaustion in HCC. P38 inhibitor attenuated the promotional effect of TGF-*β* on the expression of PD-1 and TIM-3 in CD8 + T cells. However, TGF-*β* alleviated the exhaustion of CD8 + T cells by inducing signal transducer and activator of transcription 1 (STAT1) phosphorylation, promoting leukocyte-associated immunoglobulin-like receptor 2 (LAIR2) secretion, and inhibiting "collagen-LAIR1" signal pathway, which is referred to "self-rescue," was also demonstrated in this study. However, this self-rescue behavior only occurred under short-term or low concentration of TGF-*β* exposure [62].

During the chronic virus infection, precursors of exhausted T (*T*_pex_) cells were considered to play a crucial role in the maintenance of exhausted T cells [63]. *T*_Pex_ cells had the ability of long-term proliferation and self-renewal, and mediated the continuous replenishment of exhausted effector T (*T*_ex_) cells. As the main driving force of T cells exhaustion, TGF-*β* reduced mammalian target of rapamycin (mTOR) activity, promoted mitochondrial activity, and maintained metabolic fitness of *T*_pex_ cells. The preservation of cellular metabolism enabled *T*_Pex_ cells to retain long-term function during chronic infection to sustain T cells response. Furthermore, TGF-*β* signal also inhibited the effector function of *T*_ex_ cells and the differentiation of C-X3-C motif chemokine receptor 1 (CX3CR1) + *T*_ex_ cells, while promoting the differentiation of CD101 + terminally differentiated *T*_ex_ cells [64]. Other downstream molecules of TGF-*β* had also been identified during the development of T cells exhaustion in various models of chronic infection. In the murine cytomegalovirus (MCMV) infection model, it was found that CD8 + T cells in mice salivary glands and other nonlymphoid tissues expressed a variety of molecules related to T cells exhaustion, including CD73, CD39, and PD-1. Blocking CD73 improved the function of kidney-localized T cells. However, the expression of CD73 in exhausted CD8 + T cells was activated by TGF-*β* [65]. Overexpression of miR-720 was demonstrated in T cells exhaustion during chronic HBV infection. miR-720 inhibited the proliferation of antigen-specific CD8 + T cells by targeting the expression of cell cycle regulator AP-1 transcription factor subunit (FosB) and c-Myc. Overexpression of miR-720 in T cells was trigged by TGF-*β* [66]. These results provided important insights into the role of viral antigens and TGF-*β* in causing T cells exhaustion.

Overall, the TGF-*β* signaling pathway plays a critical role in mediating CD8 + T-cell exhaustion by promoting the expression of exhaustion markers, suppressing effector functions, promoting Treg differentiation, and regulating transcriptional programs. Targeting TGF-*β* signaling pathways represents a potential therapeutic strategy for reversing CD8 + T-cell exhaustion and enhancing anti-tumor or anti-viral immunity in chronic infections or cancer.

## Clinical application of targeting CD8 + T-cell exhaustion via TGF-*β*

The use of inhibitors or antibodies against TGF-*β* represents a promising approach for both clinical and research applications, particularly in the context of CD8 + T-cell exhaustion and its associated pathologies, such as cancer. Inhibitors or antibodies targeting TGF-*β* can be used in cancer immunotherapy to overcome immunosuppressive mechanisms within the tumor microenvironment. By blocking TGF-*β* signaling, these agents may enhance the anti-tumor activity of CD8 + T cells and other immune effector cells, leading to improved tumor control and patient outcomes. TGF-*β* inhibitors can be used alone to directly target the immunosuppressive pathways within the tumor microenvironment. Examples include small molecule inhibitors, monoclonal antibodies against TGF-*β* or its receptors, and ligand traps [67]. Combining TGF-*β* inhibitors with other immunotherapies, such as checkpoint inhibitors (e.g., PD-1/PD-L1 and CTLA-4 antibodies), can enhance the overall anti-tumor immune response. This approach can overcome resistance to monotherapy and improve the efficacy of treatment [68, 69]. Engineering T cells to be resistant to TGF-*β* signaling through genetic modifications (e.g., CRISPR/Cas9-mediated knockout of TGF-*β* receptors) can enhance the effectiveness of adoptive cell therapies. These modified T cells can persist and function better in the immunosuppressive tumor microenvironment [70]. By modulating the tumor microenvironment and reversing T-cell exhaustion, TGF-*β* inhibitors can potentially improve tumor control and patient outcomes. Continued research and clinical trials are essential to optimize these therapies and fully realize their potential in cancer treatment.

## Conclusion

T cells exhaustion refers to a transcriptionally and epigenetically distinct state of CD8 + T cells differentiation, which is characterized by decreased effector function, changes in cellular persistence and homeostasis, and increased expression of inhibitory receptors. In recent years, the potential mechanisms of T cells exhaustion have been extensively studied, and the clinical successes of exhausted T cells reversal in immunotherapy are becoming more and more common [71]. Chronic antigen recognition is the main factor that triggers the differentiation of exhausted CD8 + T cells. Factors other than chronic antigens can also affect exhaustion, including cytokines such as TGF-*β*. As an important immune response regulator, the role of TGF-*β* in promoting T cells exhaustion had been reviewed in this manuscript. Despite the above findings, many fundamental questions remain unanswered. The interactions of TGF-*β* and other cytokines are complex, and further studies are needed to reveal the specific role of TGF-*β* in the formation of exhausted CD8 + T cells. Second, TGF-*β* has different effects on various immune cells in tumor microenvironment, and how TGF-*β*-treated immune cells affect the development of exhausted CD8 + T cells is unclear. Finally, both TCR signaling pathway and costimulatory receptor are regulated by TGF-*β*. The balances of these two actions on the formation of exhausted CD8 + T cells are needed to be further studied. Addressing these issues may help to introduce the next generation of T cells-based therapies and expand our understanding of T cells dysfunction in chronic diseases.

## Data Availability

Not applicable.

## Abbreviations

AP-1: Activator protein-1
BCL6: B-cell lymphoma 6
BLIMP1: B-lymphocyte-induced maturation protein 1
BM: Bone marrow
BMP: Bone morphogenetic protein
COX-2: Cyclooxygenase-2
crEVs: Circulating extracellular vesicles
CTLA-4: Cytotoxic T lymphocyte antigen 4
CX3CR1: C-X3-C motif chemokine receptor 1
ERK1/2: Extracellular signal-regulated kinase 1/2
FOXP1: Forkhead box protein 1
GARP: Glycoprotein-A repetitions predominant
GATA3: GATA-binding protein 3
HCC: Hepatocellular carcinoma
ICIs: Immune checkpoint inhibitors
IFN-*γ*: Interferon-gamma
IKKα: IκB kinase-*α*
IL-2: Interleukin-2
LAG-3: Lymphocyte activation gene-3
LAIR2: Leukocyte-associated immunoglobulin-like receptor 2
MAP: Mitogen-activated protein
MCMV: Murine cytomegalovirus
MH2: Mad homology 2
mTOR: Mammalian target of rapamycin
NFAT: Nuclear factor of activated T cells
NR4A: Nuclear receptor 4A
PD-1: Programmed death-1
STAT1: Signal transducer and activator of transcription 1
TAK1: TGF-*β*-activated kinase 1
TCF1: T cytokine 1
TCNVs: T-cell-derived nanovesicles
TCR: T-cell receptors
Term: Terminally differentiated CD8 + T
TEV: Tumor-derived extracellular vesicles
Tex: Exhausted effector T
TGF-*β*: Transforming growth factor-*β*
TGF-*β*RII: TGF-*β* receptor II
TIF1*γ*: Transcriptional intermediary factor 1 *γ*
TIGIT: T-cell immunoglobulin and ITIM domain
TIM-3: T-cell immunoglobulin and mucin domain-containing protein 3
TOX: Thymocyte selection-associated high mobility group box factor
Tpex: Precursors of exhausted T
Tprog: Progenitor-like CD8 + T
USP8: Ubiquitin-specific protease 8

## References

[1] He X, Xu C. Immune checkpoint signaling and cancer immunotherapy. Cell Res. 2020;30(8):660–9. 32467592 10.1038/s41422-020-0343-4 PMC7395714

[2] Ribas A, Wolchok JD. Cancer immunotherapy using checkpoint blockade. Science. 2018;359(6382):1350–5. 29567705 10.1126/science.aar4060 PMC7391259

[3] De Plaen E, Lurquin C, Van Pel A, Mariame B, Szikora JP, Wolfel T, Sibille C, Chomez P, Boon T. Immunogenic (tum-) variants of mouse tumor P815: cloning of the gene of tum- antigen P91A and identification of the tum- mutation. Proc Natl Acad Sci U S A. 1988;85(7):2274–8. 3127830 10.1073/pnas.85.7.2274 PMC279973

[4] Jager E, Nagata Y, Gnjatic S, Wada H, Stockert E, Karbach J, Dunbar PR, Lee SY, Jungbluth A, Jager D, et al. Monitoring CD8 T cell responses to NY-ESO-1: correlation of humoral and cellular immune responses. Proc Natl Acad Sci U S A. 2000;97(9):4760–5. 10781081 10.1073/pnas.97.9.4760 PMC18306

[5] Gros A, Robbins PF, Yao X, Li YF, Turcotte S, Tran E, Wunderlich JR, Mixon A, Farid S, Dudley ME, et al. PD-1 identifies the patient-specific CD8(+) tumor-reactive repertoire infiltrating human tumors. J Clin Invest. 2014;124(5):2246–59. 24667641 10.1172/JCI73639 PMC4001555

[6] Baitsch L, Baumgaertner P, Devevre E, Raghav SK, Legat A, Barba L, Wieckowski S, Bouzourene H, Deplancke B, Romero P, et al. Exhaustion of tumor-specific CD8(+) T cells in metastases from melanoma patients. J Clin Invest. 2011;121(6):2350–60. 21555851 10.1172/JCI46102 PMC3104769

[7] Gallimore A, Glithero A, Godkin A, Tissot AC, Pluckthun A, Elliott T, Hengartner H, Zinkernagel R. Induction and exhaustion of lymphocytic choriomeningitis virus-specific cytotoxic T lymphocytes visualized using soluble tetrameric major histocompatibility complex class I-peptide complexes. J Exp Med. 1998;187(9):1383–93. 9565631 10.1084/jem.187.9.1383 PMC2212278

[8] Moskophidis D, Lechner F, Pircher H, Zinkernagel RM. Virus persistence in acutely infected immunocompetent mice by exhaustion of antiviral cytotoxic effector T cells. Nature. 1993;362(6422):758–61. 8469287 10.1038/362758a0

[9] Budimir N, Thomas GD, Dolina JS, Salek-Ardakani S. Reversing T-cell exhaustion in cancer: lessons learned from PD-1/PD-L1 immune checkpoint blockade. Cancer Immunol Res. 2022;10(2):146–53. 34937730 10.1158/2326-6066.CIR-21-0515

[10] Miller BC, Sen DR, Al Abosy R, Bi K, Virkud YV, LaFleur MW, Yates KB, Lako A, Felt K, Naik GS, et al. Subsets of exhausted CD8(+) T cells differentially mediate tumor control and respond to checkpoint blockade. Nat Immunol. 2019;20(3):326–36. 30778252 10.1038/s41590-019-0312-6 PMC6673650

[11] Batlle E, Massague J. Transforming growth factor-beta signaling in immunity and cancer. Immunity. 2019;50(4):924–40. 30995507 10.1016/j.immuni.2019.03.024 PMC7507121

[12] Travis MA, Sheppard D. TGF-beta activation and function in immunity. Annu Rev Immunol. 2014;32:51–82. 24313777 10.1146/annurev-immunol-032713-120257 PMC4010192

[13] Yang L, Pang Y, Moses HL. TGF-beta and immune cells: an important regulatory axis in the tumor microenvironment and progression. Trends Immunol. 2010;31(6):220–7. 20538542 10.1016/j.it.2010.04.002 PMC2891151

[14] Moreau JM, Velegraki M, Bolyard C, Rosenblum MD, Li Z. Transforming growth factor-beta1 in regulatory T cell biology. Sci Immunol. 2022;7(69):eabi4613. 35302863 10.1126/sciimmunol.abi4613 PMC10552796

[15] Saadey AA, Yousif A, Osborne N, Shahinfar R, Chen YL, Laster B, Rajeev M, Bauman P, Webb A, Ghoneim HE. Rebalancing TGFbeta1/BMP signals in exhausted T cells unlocks responsiveness to immune checkpoint blockade therapy. Nat Immunol. 2023;24(2):280–94. 36543960 10.1038/s41590-022-01384-y

[16] Hu Y, Hudson WH, Kissick HT, Medina CB, Baptista AP, Ma C, Liao W, Germain RN, Turley SJ, Zhang N, et al. TGF-beta regulates the stem-like state of PD-1+ TCF-1+ virus-specific CD8 T cells during chronic infection. J Exp Med. 2022. 10.1084/jem.20211574. 35980386 10.1084/jem.20211574 PMC9393409

[17] Xie F, Zhou X, Su P, Li H, Tu Y, Du J, Pan C, Wei X, Zheng M, Jin K, et al. Breast cancer cell-derived extracellular vesicles promote CD8(+) T cell exhaustion via TGF-beta type II receptor signaling. Nat Commun. 2022;13(1):4461. 35915084 10.1038/s41467-022-31250-2 PMC9343611

[18] Hashimoto M, Kamphorst AO, Im SJ, Kissick HT, Pillai RN, Ramalingam SS, Araki K, Ahmed R. CD8 T cell exhaustion in chronic infection and cancer: opportunities for interventions. Annu Rev Med. 2018;69:301–18. 29414259 10.1146/annurev-med-012017-043208

[19] McLane LM, Abdel-Hakeem MS, Wherry EJ. CD8 T cell exhaustion during chronic viral infection and cancer. Annu Rev Immunol. 2019;37:457–95. 30676822 10.1146/annurev-immunol-041015-055318

[20] Sade-Feldman M, Yizhak K, Bjorgaard SL, Ray JP, de Boer CG, Jenkins RW, Lieb DJ, Chen JH, Frederick DT, Barzily-Rokni M, et al. Defining T cell states associated with response to checkpoint immunotherapy in melanoma. Cell. 2018;175(4):998–1013. 30388456 10.1016/j.cell.2018.10.038 PMC6641984

[21] Paley MA, Kroy DC, Odorizzi PM, Johnnidis JB, Dolfi DV, Barnett BE, Bikoff EK, Robertson EJ, Lauer GM, Reiner SL, et al. Progenitor and terminal subsets of CD8+ T cells cooperate to contain chronic viral infection. Science. 2012;338(6111):1220–5. 23197535 10.1126/science.1229620 PMC3653769

[22] Gebhardt T, Park SL, Parish IA. Stem-like exhausted and memory CD8(+) T cells in cancer. Nat Rev Cancer. 2023;23(11):780–98. 37821656 10.1038/s41568-023-00615-0

[23] van der Leun AM, Thommen DS, Schumacher TN. CD8(+) T cell states in human cancer: insights from single-cell analysis. Nat Rev Cancer. 2020;20(4):218–32. 32024970 10.1038/s41568-019-0235-4 PMC7115982

[24] Wherry EJ, Kurachi M. Molecular and cellular insights into T cell exhaustion. Nat Rev Immunol. 2015;15(8):486–99. 26205583 10.1038/nri3862 PMC4889009

[25] Franco F, Jaccard A, Romero P, Yu YR, Ho PC. Metabolic and epigenetic regulation of T-cell exhaustion. Nat Metab. 2020;2(10):1001–12. 32958939 10.1038/s42255-020-00280-9

[26] Martinez GJ, Pereira RM, Aijo T, Kim EY, Marangoni F, Pipkin ME, Togher S, Heissmeyer V, Zhang YC, Crotty S, et al. The transcription factor NFAT promotes exhaustion of activated CD8(+) T cells. Immunity. 2015;42(2):265–78. 25680272 10.1016/j.immuni.2015.01.006 PMC4346317

[27] Alfei F, Kanev K, Hofmann M, Wu M, Ghoneim HE, Roelli P, Utzschneider DT, von Hoesslin M, Cullen JG, Fan Y, et al. TOX reinforces the phenotype and longevity of exhausted T cells in chronic viral infection. Nature. 2019;571(7764):265–9. 31207605 10.1038/s41586-019-1326-9

[28] Scott AC, Dundar F, Zumbo P, Chandran SS, Klebanoff CA, Shakiba M, Trivedi P, Menocal L, Appleby H, Camara S, et al. TOX is a critical regulator of tumour-specific T cell differentiation. Nature. 2019;571(7764):270–4. 31207604 10.1038/s41586-019-1324-y PMC7698992

[29] Chen J, Lopez-Moyado IF, Seo H, Lio CJ, Hempleman LJ, Sekiya T, Yoshimura A, Scott-Browne JP, Rao A. NR4A transcription factors limit CAR T cell function in solid tumours. Nature. 2019;567(7749):530–4. 30814732 10.1038/s41586-019-0985-x PMC6546093

[30] Blackburn SD, Shin H, Haining WN, Zou T, Workman CJ, Polley A, Betts MR, Freeman GJ, Vignali DA, Wherry EJ. Coregulation of CD8+ T cell exhaustion by multiple inhibitory receptors during chronic viral infection. Nat Immunol. 2009;10(1):29–37. 19043418 10.1038/ni.1679 PMC2605166

[31] Araki K, Youngblood B, Ahmed R. Programmed cell death 1-directed immunotherapy for enhancing T-cell function. Cold Spring Harb Symp Quant Biol. 2013;78:239–47. 25028401 10.1101/sqb.2013.78.019869

[32] Okagawa T, Konnai S, Nishimori A, Maekawa N, Goto S, Ikebuchi R, Kohara J, Suzuki Y, Yamada S, Kato Y, et al. Cooperation of PD-1 and LAG-3 in the exhaustion of CD4(+) and CD8(+) T cells during bovine leukemia virus infection. Vet Res. 2018;49(1):50. 29914540 10.1186/s13567-018-0543-9 PMC6006750

[33] Chiu DK, Yuen VW, Cheu JW, Wei LL, Ting V, Fehlings M, Sumatoh H, Nardin A, Newell EW, Ng IO, et al. Hepatocellular carcinoma cells up-regulate PVRL1, stabilizing PVR and inhibiting the cytotoxic T-cell response via TIGIT to mediate tumor resistance to PD1 inhibitors in mice. Gastroenterology. 2020;159(2):609–23. 32275969 10.1053/j.gastro.2020.03.074

[34] David CJ, Massague J. Contextual determinants of TGFbeta action in development, immunity and cancer. Nat Rev Mol Cell Biol. 2018;19(7):419–35. 29643418 10.1038/s41580-018-0007-0 PMC7457231

[35] Li MO, Wan YY, Sanjabi S, Robertson AK, Flavell RA. Transforming growth factor-beta regulation of immune responses. Annu Rev Immunol. 2006;24:99–146. 16551245 10.1146/annurev.immunol.24.021605.090737

[36] Ouyang W, Beckett O, Ma Q, Li MO. Transforming growth factor-beta signaling curbs thymic negative selection promoting regulatory T cell development. Immunity. 2010;32(5):642–53. 20471291 10.1016/j.immuni.2010.04.012 PMC2880228

[37] Shull MM, Ormsby I, Kier AB, Pawlowski S, Diebold RJ, Yin M, Allen R, Sidman C, Proetzel G, Calvin D, et al. Targeted disruption of the mouse transforming growth factor-beta 1 gene results in multifocal inflammatory disease. Nature. 1992;359(6397):693–9. 1436033 10.1038/359693a0 PMC3889166

[38] Kulkarni AB, Huh CG, Becker D, Geiser A, Lyght M, Flanders KC, Roberts AB, Sporn MB, Ward JM, Karlsson S. Transforming growth factor beta 1 null mutation in mice causes excessive inflammatory response and early death. Proc Natl Acad Sci U S A. 1993;90(2):770–4. 8421714 10.1073/pnas.90.2.770 PMC45747

[39] Hinck AP, Mueller TD, Springer TA. Structural biology and evolution of the TGF-beta family. Cold Spring Harb Perspect Biol. 2016;8(12):a022103. 27638177 10.1101/cshperspect.a022103 PMC5131774

[40] Shi Y, Massague J. Mechanisms of TGF-beta signaling from cell membrane to the nucleus. Cell. 2003;113(6):685–700. 12809600 10.1016/s0092-8674(03)00432-x

[41] Zhang YE. Non-Smad pathways in TGF-beta signaling. Cell Res. 2009;19(1):128–39. 19114990 10.1038/cr.2008.328 PMC2635127

[42] Li MO, Sanjabi S, Flavell RA. Transforming growth factor-beta controls development, homeostasis, and tolerance of T cells by regulatory T cell-dependent and -independent mechanisms. Immunity. 2006;25(3):455–71. 16973386 10.1016/j.immuni.2006.07.011

[43] Ma C, Zhang N. Lymphoid tissue residency: a key to understand Tcf-1(+)PD-1(+) T cells. Front Immunol. 2022;13:1074698. 36569850 10.3389/fimmu.2022.1074698 PMC9767944

[44] Lyu X, Li G, Qiao Q. Identification of an immune classification for cervical cancer and integrative analysis of multiomics data. J Transl Med. 2021;19(1):200. 33971902 10.1186/s12967-021-02845-y PMC8111986

[45] Hartana CA, Ahlen Bergman E, Zirakzadeh AA, Krantz D, Winerdal ME, Winerdal M, Johansson M, Alamdari F, Jakubczyk T, Glise H, et al. Urothelial bladder cancer may suppress perforin expression in CD8+ T cells by an ICAM-1/TGFbeta2 mediated pathway. PLoS ONE. 2018;13(7):e0200079. 29966014 10.1371/journal.pone.0200079 PMC6028111

[46] Zidan M, Zidan AA, Attia Saad M, El-Shanshory M, Bakry U, Sobh A, Mohammed Abdou S, Labib SM. Altered microRNA expression profile is linked to T-cell exhaustion-related pathways in pediatric patients with acute lymphoblastic leukemia. Hum Immunol. 2023;84(2):113–22. 36347735 10.1016/j.humimm.2022.10.005

[47] Staudacher JJ, Bauer J, Jana A, Tian J, Carroll T, Mancinelli G, Ozden O, Krett N, Guzman G, Kerr D, et al. Activin signaling is an essential component of the TGF-beta induced pro-metastatic phenotype in colorectal cancer. Sci Rep. 2017;7(1):5569. 28717230 10.1038/s41598-017-05907-8 PMC5514149

[48] Wiley MB, Bauer J, Mehrotra K, Zessner-Spitzenberg J, Kolics Z, Cheng W, Castellanos K, Nash MG, Gui X, Kone L, et al. Non-canonical Activin A signaling stimulates context-dependent and cellular-specific outcomes in CRC to promote tumor cell migration and immune tolerance. Cancers (Basel). 2023;15(11):3003. 37296966 10.3390/cancers15113003 PMC10252122

[49] Kwon M, Kim CG, Lee H, Cho H, Kim Y, Lee EC, Choi SJ, Park J, Seo IH, Bogen B, et al. PD-1 blockade reinvigorates bone marrow CD8(+) T cells from patients with multiple myeloma in the presence of TGFbeta inhibitors. Clin Cancer Res. 2020;26(7):1644–55. 31941832 10.1158/1078-0432.CCR-19-0267

[50] Hong J, Kang M, Jung M, Lee YY, Cho Y, Kim C, Song SY, Park CG, Doh J, Kim BS. T-cell-derived nanovesicles for cancer immunotherapy. Adv Mater. 2021;33(33):e2101110. 34235790 10.1002/adma.202101110

[51] Li A, Chang Y, Song NJ, Wu X, Chung D, Riesenberg BP, Velegraki M, Giuliani GD, Das K, Okimoto T, et al. Selective targeting of GARP-LTGFbeta axis in the tumor microenvironment augments PD-1 blockade via enhancing CD8(+) T cell antitumor immunity. J Immunother Cancer. 2022;10(9):e005433. 36096533 10.1136/jitc-2022-005433 PMC9472209

[52] Rej A, Paladhi A, Daripa S, Sarkar D, Bhattacharyya S, Mondal I, Hira SK. Galunisertib synergistically potentiates the doxorubicin-mediated antitumor effect and kickstarts the immune system against aggressive lymphoma. Int Immunopharmacol. 2023;114:109521. 36470118 10.1016/j.intimp.2022.109521

[53] Sievers C, Craveiro M, Friedman J, Robbins Y, Yang X, Bai K, Nguyen A, Redman JM, Chari R, Soon-Shiong P, et al. Phenotypic plasticity and reduced tissue retention of exhausted tumor-infiltrating T cells following neoadjuvant immunotherapy in head and neck cancer. Cancer Cell. 2023;41(5):887–902. 37059104 10.1016/j.ccell.2023.03.014 PMC10175181

[54] Tinoco R, Alcalde V, Yang Y, Sauer K, Zuniga EI. Cell-intrinsic transforming growth factor-beta signaling mediates virus-specific CD8+ T cell deletion and viral persistence in vivo. Immunity. 2009;31(1):145–57. 19604493 10.1016/j.immuni.2009.06.015 PMC3039716

[55] Lin DY, Tanaka Y, Iwasaki M, Gittis AG, Su HP, Mikami B, Okazaki T, Honjo T, Minato N, Garboczi DN. The PD-1/PD-L1 complex resembles the antigen-binding Fv domains of antibodies and T cell receptors. Proc Natl Acad Sci U S A. 2008;105(8):3011–6. 18287011 10.1073/pnas.0712278105 PMC2268576

[56] Chatterjee S, Chatterjee A, Jana S, Dey S, Roy H, Das MK, Alam J, Adhikary A, Chowdhury A, Biswas A, et al. Transforming growth factor beta orchestrates PD-L1 enrichment in tumor-derived exosomes and mediates CD8 T-cell dysfunction regulating early phosphorylation of TCR signalome in breast cancer. Carcinogenesis. 2021;42(1):38–47. 32832992 10.1093/carcin/bgaa092

[57] Xie F, Zhou X, Li H, Su P, Liu S, Li R, Zou J, Wei X, Pan C, Zhang Z, et al. USP8 promotes cancer progression and extracellular vesicle-mediated CD8+ T cell exhaustion by deubiquitinating the TGF-beta receptor TbetaRII. EMBO J. 2022;41(16):e108791. 35811497 10.15252/embj.2021108791 PMC9379553

[58] Takahashi S. Functional analysis of large MAF transcription factors and elucidation of their relationships with human diseases. Exp Anim. 2021;70(3):264–71. 33762508 10.1538/expanim.21-0027 PMC8390310

[59] Giordano M, Henin C, Maurizio J, Imbratta C, Bourdely P, Buferne M, Baitsch L, Vanhille L, Sieweke MH, Speiser DE, et al. Molecular profiling of CD8 T cells in autochthonous melanoma identifies Maf as driver of exhaustion. EMBO J. 2015;34(15):2042–58. 26139534 10.15252/embj.201490786 PMC4551351

[60] Sun Q, Cai D, Liu D, Zhao X, Li R, Xu W, Xie B, Gou M, Wei K, Li Y, et al. BCL6 promotes a stem-like CD8(+) T cell program in cancer via antagonizing BLIMP1. Sci Immunol. 2023;8(88):eadh1306. 37862431 10.1126/sciimmunol.adh1306

[61] Xun X, Zhang C, Wang S, Hu S, Xiang X, Cheng Q, Li Z, Wang Y, Zhu J. Cyclooxygenase-2 expressed hepatocellular carcinoma induces cytotoxic T lymphocytes exhaustion through M2 macrophage polarization. Am J Transl Res. 2021;13(5):4360–75. 34150019 PMC8205841

[62] Pan B, Wang Z, Yao Y, Ke X, Shen S, Chen W, Zhang X, Qiu J, Wu X, Tang N. TGF-beta-p-STAT1-LAIR2 axis has a “self-rescue” role for exhausted CD8(+) T cells in hepatocellular carcinoma. Cell Oncol (Dordr). 2023;46(6):1625–44. 37223874 10.1007/s13402-023-00830-9 PMC12974680

[63] Kallies A, Zehn D, Utzschneider DT. Precursor exhausted T cells: key to successful immunotherapy? Nat Rev Immunol. 2020;20(2):128–36. 31591533 10.1038/s41577-019-0223-7

[64] Gabriel SS, Tsui C, Chisanga D, Weber F, Llano-Leon M, Gubser PM, Bartholin L, Souza-Fonseca-Guimaraes F, Huntington ND, Shi W, et al. Transforming growth factor-beta-regulated mTOR activity preserves cellular metabolism to maintain long-term T cell responses in chronic infection. Immunity. 2021;54(8):1698–714. 34233154 10.1016/j.immuni.2021.06.007

[65] Smith CJ, Snyder CM. Inhibitory molecules PD-1, CD73 and CD39 are expressed by CD8(+) T cells in a tissue-dependent manner and can inhibit T cell responses to stimulation. Front Immunol. 2021;12:704862. 34335618 10.3389/fimmu.2021.704862 PMC8320728

[66] Wang Y, Zhang Z, Ji D, Chen GF, Feng X, Gong LL, Guo J, Li ZW, Chen CF, Zhao BB, et al. Regulation of T cell function by microRNA-720. Sci Rep. 2015;5:12159. 26199080 10.1038/srep12159 PMC4510490

[67] van den Bulk J, de Miranda N, Ten Dijke P. Therapeutic targeting of TGF-beta in cancer: hacking a master switch of immune suppression. Clin Sci (Lond). 2021;135(1):35–52. 33399850 10.1042/CS20201236 PMC7796313

[68] Mariathasan S, Turley SJ, Nickles D, Castiglioni A, Yuen K, Wang Y, Kadel EE III, Koeppen H, Astarita JL, Cubas R, et al. TGFbeta attenuates tumour response to PD-L1 blockade by contributing to exclusion of T cells. Nature. 2018;554(7693):544–8. 29443960 10.1038/nature25501 PMC6028240

[69] Li T, Wang X, Niu M, Wang M, Zhou J, Wu K, Yi M. Bispecific antibody targeting TGF-beta and PD-L1 for synergistic cancer immunotherapy. Front Immunol. 2023;14:1196970. 37520520 10.3389/fimmu.2023.1196970 PMC10373067

[70] Fix SM, Forget MA, Sakellariou-Thompson D, Wang Y, Griffiths TM, Lee M, Haymaker CL, Dominguez AL, Basar R, Reyes C, et al. CRISPR-mediated TGFBR2 knockout renders human ovarian cancer tumor-infiltrating lymphocytes resistant to TGF-beta signaling. J Immunother Cancer. 2022;10(7):e003750. 35882447 10.1136/jitc-2021-003750 PMC9330322

[71] Chow A, Perica K, Klebanoff CA, Wolchok JD. Clinical implications of T cell exhaustion for cancer immunotherapy. Nat Rev Clin Oncol. 2022;19(12):775–90. 36216928 10.1038/s41571-022-00689-z PMC10984554

